# Synchrotron radiation computed laminography for polymer composite failure studies

**DOI:** 10.1107/S0909049510001512

**Published:** 2010-01-30

**Authors:** Feng Xu, Lukas Helfen, Andrew J. Moffat, Gregory Johnson, Ian Sinclair, Tilo Baumbach

**Affiliations:** a ANKA/Institute for Synchrotron Radiation, Karlsruhe Institute of Technology, Germany; b School of Engineering Sciences, University of Southampton, UK; c European Synchrotron Radiation Facility, Grenoble, France

**Keywords:** laminography, *in situ* loading, polymer composites, phase retrieval

## Abstract

Synchrotron radiation computed laminography is applied to the three-dimensional micro-imaging of damage in large polymer composite plates with high spatial resolution. The influence of different experimental conditions is studied with respect to measurement time optimization, dose minimization and reduction of artefacts in the reconstructed images. Failures like delaminations, transverse ply cracks and splits are observed under *in situ* loads. The propagation of up to 2 mm-long cracks is non-destructively followed *in situ* and investigated in detail. By phase retrieval using a single detector distance, the failures can be easily visualized in three dimensions.

## Abstract 2

Failures of laterally extended polymer composite panels are imaged using 3D computed laminography. The experimental parameters and capability of the method are studied.

## 1. Introduction

Computed laminography (CL) using synchrotron radiation (SR) has been developed as a non-destructive three-dimensional (3D) imaging method for flat specimens (Helfen *et al.*, 2005 ▶). Compared with its laboratory counterparts, the use of synchrotron radiation provides high flux and an almost parallel X-ray beam enabling fast and monochromatic imaging. Moreover, qualitative and quantitative phase-contrast imaging becomes possible (Nugent *et al.*, 1996 ▶; Cloetens *et al.*, 1999 ▶, 2002 ▶; Paganin *et al.*, 2002 ▶) which has been extensively shown in computed tomography (CT), a 3D imaging technique nowadays well established at synchrotron imaging set-ups.

SR-CL enables high-resolution 3D imaging of regions of interest (ROIs) in laterally extended specimens. Although SR-CL has been mostly motivated by and developed for non-destructive micro-system device inspection (Helfen *et al.*, 2006 ▶, 2007 ▶) with high spatial resolution, other application fields start to emerge which profit from additional advantages of SR imaging. For example, exploiting the partial coherence of the synchrotron beam for phase-contrast laminography (Helfen *et al.*, 2009 ▶), applications in cultural heritage, *e.g.* where weakly absorbing structures in the neighbourhood of highly absorbing paint layers have to be imaged (Krug *et al.*, 2008 ▶), become feasible.

In this paper, we will demonstrate the applicability and the high potential of SR-CL in a new application field, the *in situ* study of damage in polymer composites at engineering length-scales. Polymer composites are increasingly exploited in automotive and aerospace development and other transportations, as well as in defence research owing to their light weight and high strength. Motivated by the performance parameters required for their use in those application fields there is a fundamental interest in investigation of damage evolution, which is a four-dimensional phenomenon. None of the methods used recently, *e.g.* acoustic emission sensing (Sihn *et al.*, 2007 ▶), optical fibre sensing techniques (Yashiro *et al.*, 2007 ▶), thermography (Emery & Dulieu-Barton, 2007 ▶), is able to observe the damage mechanisms directly in three dimensions for such composite materials.

On first view, CT seems to be a non-destructive solution (Moffat *et al.*, 2008 ▶). However, owing to the limited pixel number in detectors, it requires the specimen to be small enough to fit to the field of view. Otherwise, when a laterally extended object strongly exceeds the lateral field of view of the detector (as is typically the case for conventional structural testing coupons), CT suffers from exceedingly high absorption (and resulting artefacts from insufficient transmission) when the plate-like object is near to parallel to the beam direction. To avoid this results in a destructive method since a specimen with the ROI containing the damage structure has to be extracted from a mechanically representative loading state. Using SR-CL, the ROI can be imaged without specimen extraction for laterally extended specimens. This allows the application of external loads and simultaneous micro-imaging of the strained regions, enabling highly informative *in situ* studies of the complex and interacting failure process characteristics of these materials.

In this article, we demonstrate that laminographic imaging with SR is a promising technique to study failures in carbon composite materials which allows us to follow the damage evolution for increasing loading states. In the following, we will give a brief introduction to the principles of SR-CL. Since synchrotron beam time is usually rather limited, the experimental parameters in the imaging process were examined and optimized with respect to image quality, measurement time and doses delivered to the specimen during the experiment. Employing the partial coherence of the synchrotron beam, phase-contrast laminography was applied. The application to micro-imaging of the formation and propagation of damage in a 70 × 60 × 1 mm polymer composite panel under different *in situ* loads demonstrates the potential of the method in materials science.

## 2. Method and samples

**Figure 1:**
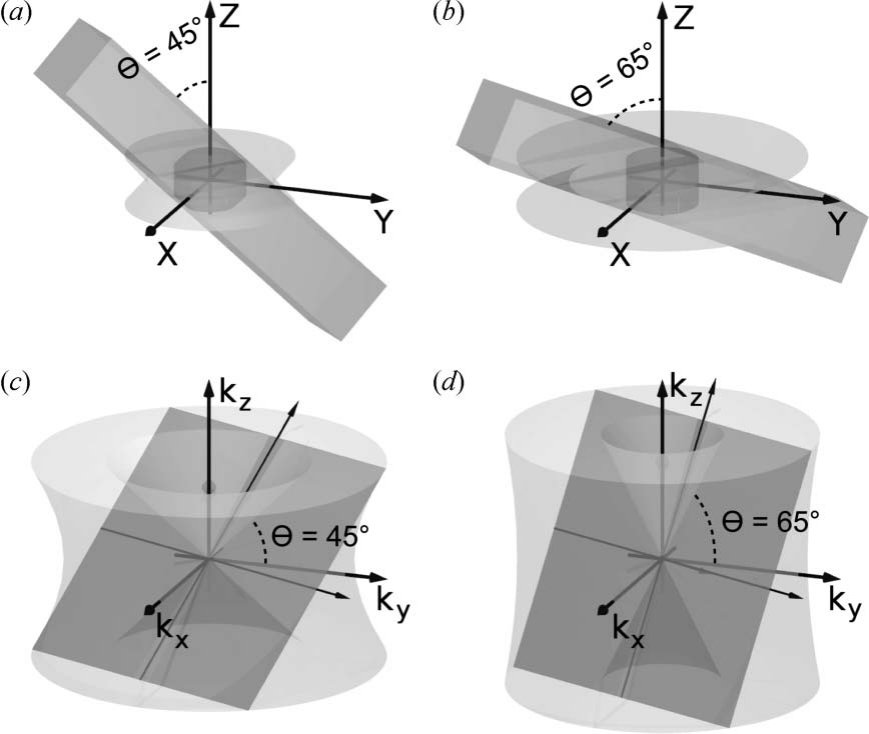
Projection and scanning geometry of SR-CL in real space (*a*) and (*b*) for θ = 45° and θ = 65°, respectively, where θ is the angle between the rotation axis *z* and the incoming beam. The dark grey bars illustrate the transmitted beam for one projection angle. (*c*) and (*d*) show the Fourier domain sampling corresponding to (*a*) and (*b*). The dark grey planes correspond to one projection in the Fourier domain. After 360° rotation of the specimen, they form the light grey parts of the Fourier domain.

In comparison with SR-CT, SR-CL is based on the inclination of the tomographic rotation axis with respect to the incident X-ray beam by the so-called laminographic angle θ [see Figs. 1(*a*) and 1(*b*) ▶]. With a flat specimen aligned approximately perpendicular to the rotation axis, the effective thickness of the specimen seen by the transmitting X-rays does not change significantly during the 360° laminographic scan around the rotation axis. During the 360° scan, the selected ROI of the specimen which is illuminated by all projection angles is illustrated in dark grey.

Laminography performs only incomplete sampling of the Fourier space, giving rise to characteristic imaging artefacts: according to the projection theorem generalized to three spatial dimensions (Dobbins & Godfrey, 2003 ▶), a single two-dimensional projection fills in Fourier space a plane perpendicular to the incoming beam direction [illustrated as a tilted dark grey plane in Figs. 1(*c*) and 1(*d*) ▶]. The rotation of the specimen fills the Fourier domain successively with data (light grey). However, owing to the inclined tomographic axis of CL, information is not collected in two cones around that axis, above and below the origin of Fourier space, as shown in Figs. 1(*c*) and 1(*d*) ▶ for θ = 45° and θ = 65°, respectively.

The laminographic angle θ between the rotation axis and the incoming beam determines the extent of the unsampled region, θ = 90° and θ = 0° being the limiting cases of computed tomography (which, however, is not applicable owing to the specimen geometry) and single direction radiography. The larger the value of θ, the smaller the unsampled regions in the Fourier domain become, see Figs. 1(*c*) and 1(*d*) ▶. The characteristic symmetry of the inaccessible region in Fourier space influences the achievable spatial resolution of detail, which therefore depends on the specimen structure itself (Helfen *et al.*, 2005 ▶). Although the missing information can cause artefacts in reconstructed images, previous CL work has given valuable results in a variety of contexts (Helfen *et al.*, 2006 ▶, 2007 ▶, 2009 ▶; Krug *et al.*, 2008 ▶; Moffat *et al.*, 2010 ▶).

Experiments were carried out on the 145 m-long beamline ID19 at the European Synchrotron Radiation Facility (ESRF) using a monochromatic X-ray beam at an energy of 20 keV. A detector based on a charge-coupled device (CCD) [the ESRF in-house development Frelon 2k (Labiche *et al.*, 2007 ▶)] with a 2048 × 2048 pixel array provided a pixel size of 0.7 µm by use of an optical lens coupling to a thin-film crystal scintillator (Martin & Koch, 2006 ▶) and a square field of view of 1.4 × 1.4 mm. 3D data sets with an isotropic voxel size of 0.7 µm were reconstructed using a filtered back-projection algorithm under various combinations of experimental parameters. Phase contrast is necessary to distinguish between the polymer matrix and carbon fibres owing to their low contrast in absorption. To facilitate 3D segmentation of the crack structure by imaging the 3D distribution of the refractive index decrement, one could apply holographic laminography (Helfen *et al.*, 2009 ▶), using phase retrieval from several specimen–detector distances. However, in order to limit the X-ray dose delivered to the specimen ROI we employed phase retrieval using a single detector distance (Paganin *et al.*, 2002 ▶) instead.

The material investigated in this work is a commercial pre-preg, carbon-fibre–epoxy unidirectional composite (Hexcel HexPly M21), with a ply thickness of approximately 250 µm. A [90/0]s laminate was produced using the manufacturer’s standard consolidation process, resulting in a total specimen thickness of approximately 1 mm.

## 3. Results and discussion

The inclination angle between rotation axis and X-ray beam is an additional experimental parameter (compared with CT) which has to be adapted to the specimen geometry and structure. Additionally we explore the influence of the number of projections and exposure time per projection on the image quality.

**Figure 2:**
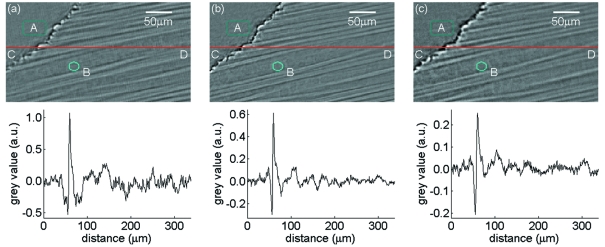
The same slice from 1500 radiographs, 200 ms exposure time, at (*a*) θ = 65°, step-wise scan; (*b*) θ = 45°, step-wise scan; (*c*) θ = 45°, continuous scan. Region *A* is air, region *B* is a resin-rich area. The plots correspond to the image values along the horizontal red lines CD.

As discussed before, the axis inclination angle plays an important role with respect to Fourier domain sampling. Two scans at θ = 65° and 45° were made on the same specimen (a test coupon pre-loaded by a 0.6 *a*/*W* notch) to clarify its effect, keeping other experimental parameters the same. After reconstruction, typical cross-sectional slices which are parallel to the rotation axis (containing both specimen and air) were carefully selected from approximately the same position (illuminated by all projections) of the notch tip region by use of normalized cross correlation [Figs. 2(*a*) (θ = 65°) and 2(*b*) (θ = 45°) ▶]. Taken from the red lines CD, reconstructed greyscale values are also plotted (see Fig. 2 ▶). The contrast is greater in Fig. 2(*a*) ▶; however, the artefacts also appear more pronounced. To quantify this, standard deviations (Std) were measured over two homogeneous regions (where no specimen structures are present) of air, *A*, and a resin-rich region, *B*. As the selected slices were acquired under otherwise the same experimental conditions, Std is suitable to outline the difference in the image quality. The Std of *A* and *B* in Fig. 2(*a*) ▶ are 0.05 and 0.06, respectively, whilst the Std of *A* and *B* in Fig. 2(*b*) ▶ are both 0.02. This seems puzzling since theory predicts less imaging artefacts (*cf.* Fig. 1 ▶) for larger θ angles. Slices perpendicular to the rotation axis show the same tendency with respect to the Std. This unusual behaviour can be explained by consideration of the specimen volumes in real space which are ‘volume 1’ illuminated for all projection angles and ‘volume 2’ not illuminated for every projection angle; see Figs. 1(*a*) and 1(*b*) ▶, dark grey for volume 1 and light grey for volume 2. For a fixed specimen thickness, volume 2 covers a comparatively larger region of the specimen for the case of the bigger laminographic angle θ = 65° than for θ = 45°. In other words, even when we are only interested in a region of volume 1, more artefacts will arise for volume 1 from the parts of the illuminated ROI (volume 2) which move in and out of the beam during the scan. So, for larger values of θ the artefacts display stronger. This has some analogy with CT with truncated projection data (sometimes called local tomography) where the artefacts depend on the size of the truncated part of the projections (*i.e.* beam width minus the specimen diameter in the case of cylindrical specimen geometry).

With the restriction of limited synchrotron beam time available, one is tempted to speed up experiments as much as possible. One solution is to employ continuous scanning (*i.e.* performing the exposure during specimen rotation) instead of step-wise scanning (where for each exposure the specimen rotation is stopped). Hence, acceleration and deceleration of the rotation stage are avoided during continuous scans. The principal disadvantage is that, depending on the integration angle under continuous rotation, the object structures not exactly on the rotation centre will become blurred, which could increase overall artefacts levels. As an example from our experiments, when taking 1500 angularly equidistant radiographs at 200 ms exposure time, a step-wise scan [see Figs. 2(*a*) for θ = 65° and 2(*b*) ▶ for θ = 45° ▶] will take around 80 min whilst a continuous scan [see Fig. 2(*c*) for θ = 45° ▶] only takes about 15 min. The Std of *A* and *B* in Fig. 2(*c*) ▶ are 0.01 and 0.02, little different from those of Fig. 2(*b*) ▶. The reconstructed values along the red line CD in Figs. 2(*a*), 2(*b*) and 2(*c*) ▶ are plotted to give an indication of the signal-to-noise ratio. From the plots and the images, taking both noise/artefacts and contrast into account, the step-wise scan at θ = 45° in Fig. 2(*b*) ▶ clearly displays the best image quality.

Other important parameters which are known from CT to determine the quality of the reconstructed image are the exposure time and number of projections acquired from the specimen (Faulkner & Moores, 1984 ▶; Supanich *et al.*, 2009 ▶). Both affect the total counting time and hence the experiment time and the doses delivered to the specimen.

**Figure 3:**
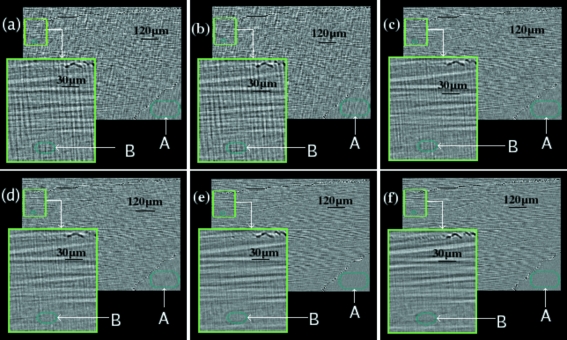
Step-wise scans with a laminographic angle θ of 65° of one specimen with different experimental parameters listed in Table 1 ▶. Region *A* is air, region *B* is a resin-rich area.

To investigate their effect in the case of SR-CL applied to polymer composite studies, a series of step-wise scans were performed at the same ROI of the same specimen with different exposure times and number of projections. The laminographic angle was set to 65°. Another specimen was treated by the notch in the same way as the previous specimen and scanned at a region close to the notch tip. Fig. 3 ▶ shows the same reconstructed slices (parallel to the rotation axis) under different experimental parameters, as listed in Table 1 ▶. The lower left squares are the zoom of the small squares in the upper left region. In all Figs. 3(*a*)–3(*f*) ▶ cracks are visible. As expected, the image quality differs for different imaging conditions. Two typical homogeneous regions (where no specimen structures are present) containing air and resin only were selected, regions *A* and *B*, respectively. The standard deviations are listed in Table 1 ▶. For similar scanning time, from the quantified values, more projections clearly yield a better quality. For example, the total illumination time of Fig. 3(*b*) ▶ is longer than that of Fig. 3(*c*) ▶, but the standard deviation is greater indicating that the reconstruction quality is poorer.

**Figure 4:**
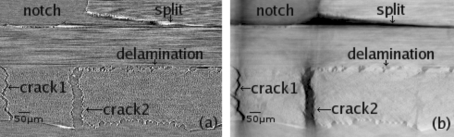
The same cross-sectional slice under *in situ* load reconstructed by SR-CL (θ = 65°) for the edge-detection regime (*a*) and applying phase retrieval from a single detector distance (*b*).

**Figure 5:**
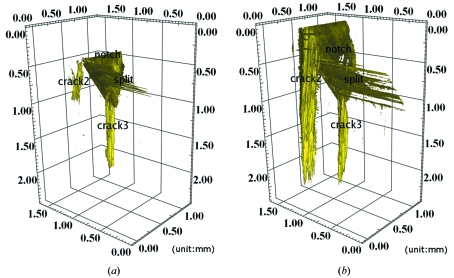
Employing phase retrieval from a single detector distance to improve the image segmentation, two 3D renditions (*a*) and (*b*) show the widening of the crack under increasing *in situ* loads.

For many load-bearing material structures, the characteristic length scale of fracture from cracks and notches can be in the centimetre range and above (Wisnom, 1999 ▶). Aiming to check the capability of SR-CL, 70 × 60 × 1 mm panels of composite were subjected to progressive damage development from a deep notch, with fracture mechanisms around the notch tip being imaged *in situ* *via* SR-CL. A displacement-controlled wedging device was used for *in situ* loading. A simple anti-buckling device, containing an aperture to allow X-ray transmission to the ROI, was used to ensure macroscopic mode loading. Scans were made close to the notch tip region. Fig. 4(*a*) ▶ shows that a split initiated and propagated away from the notch. Transverse ply cracks were observed and delaminations also occurred. In order to improve the segmentation quality and better visualize these structures in 3D, phase retrieval using a single detector distance (Paganin *et al.*, 2002 ▶) was applied for reconstruction. This computationally efficient phase-retrieval method assumes a homogeneous object and rather short specimen–detector distances. It can extract phase information by use of a transport-of-intensity equation (Rytov *et al.*, 1989 ▶). In comparison with the reconstruction in the edge-detection regime, the same slice of the reconstructed volume with phase retrieval is shown in Fig. 4(*b*) ▶. From these reconstructions involving phase retrieval, the cracks and splits under different loads could be easily extracted (simple thresholding for binarization) in the 3D renderings of Fig. 5 ▶. We see that with increasing load it is mainly the split and crack 2 which are growing; crack 3 stays almost at the same size. The split can be seen to propagate along a 45° direction owing to the notch’s shape from manufacturing. Cracks in Fig. 5(*b*) ▶ are at a length scale of 2 mm, which is larger than the field of view of the detector. Therefore, a raster scan was employed across different lateral positions (adjacent ROIs) which were concatenated afterwards by 3D image correlation techniques. As we can see, the entire crack can be three-dimensionally visualized and examined, across scales and in engineering-relevant coupon sizes that would be inaccessible with a comparable CT configuration.

## 4. Conclusions

In conclusion, it has been demonstrated that SR-CL can be applied to image ROIs in panels of polymer composites in three dimensions. Phase-contrast imaging with single detector distance phase retrieval proved effective for easy segmentation of the damage. As a result, damage could be identified and interpreted. For large growing cracks, this can be performed by combining one or more scans into one large 3D image which contains the entire damaged region, whilst maintaining high spatial resolution. Consequently, during *in situ* loading it is possible to follow the damage propagation over a wide range of loads. This study demonstrates that SR-CL opens up significant practical opportunities for understanding the four-dimensional phenomenon of damage evolution and its mechanisms in a wide range of composite materials.

Furthermore, the relevant parameters space of SR-CL was examined for the study of such composite materials. It was shown that for the particular specimen thickness and spatial resolution (determining the field of view of the detector) chosen, a laminographic angle of 45° offers better overall reconstruction quality than 65° and reasons for this were given. For further *in situ* studies and relatively thick composite panels (in comparison with the detector’s field of view), an axis inclination angle of 45° and step-wise scanning with a large number of projections (of the order of 1500) at low exposure time led us to expect even better imaging quality than shown in Figs. 4 ▶ and 5 ▶.

## Figures and Tables

**Table 1:** Experimental parameters and quantified values of Fig. 3 ▶

	(*a*)	(*b*)	(*c*)	(*d*)	(*e*)	(*f*)
Exposure time (ms)	50	100	50	100	100	200
Number of projections	600	600	1000	1000	1500	1500
Std of region *A*	0.10	0.10	0.06	0.06	0.04	0.03
Std of region *B*	0.16	0.16	0.11	0.08	0.07	0.07
